# Neoadjuvant therapy in pancreatic cancer: a systematic review and meta‐analysis of prospective studies

**DOI:** 10.1002/cam4.1071

**Published:** 2017-05-23

**Authors:** Han‐Xiang Zhan, Jian‐Wei Xu, Dong Wu, Zhi‐Yang Wu, Lei Wang, San‐Yuan Hu, Guang‐Yong Zhang

**Affiliations:** ^1^ Department of General SurgeryQilu hospitalShandong UniversityJinanShandong Province250012China

**Keywords:** Neoadjuvant therapy, pancreatic cancer, R0 resection, survival, tumor response

## Abstract

There is a strong rationale and many theoretical advantages for neoadjuvant therapy in pancreatic cancer (PC). However, study results have varied significantly. In this study, a systematic review and meta‐analysis of prospective studies were performed in order to evaluate safety and effectiveness of neoadjuvant therapy in PC. Thirty‐nine studies were selected (*n *= 1458 patients), with 14 studies focusing on patients with resectable disease (group 1), and 19 studies focusing on patients with borderline resectable and locally advanced disease (group 2). Neoadjuvant chemotherapy was administered in 97.4% of the studies, in which 76.9% was given radiotherapy and 74.4% administered with chemoradiation. The complete and partial response rate was 3.8% and 20.9%. The incidence of grade 3/4 toxicity was 11.3%. The overall resection rate after neoadjuvant therapy was 57.7% (group 1: 73.0%, group 2: 40.2%). The R0 resection rate was 84.2% (group 1: 88.2%, group 2: 79.4%). The overall survival for all patients was 16.79 months (resected 24.24, unresected 9.81; group 1: 17.76, group 2: 16.20). Our results demonstrate that neoadjuvant therapy has not been proven to be beneficial and should be considered with caution in patients with resectable PC. Patients with borderline resectable or locally advanced disease may benefit from neoadjuvant therapy, but further research is needed.

## Introduction

Despite advances in the understanding of pancreatic cancer (PC) carcinogenesis and therapeutic agents, PC remains one of the most lethal malignancies, with an overall 5‐year survival rate of approximately 5% 1, 2. Radical resection with a negative margin (R0 resection) is the key factor for long‐term survival of this aggressive malignancy 3, 4. Unfortunately, due to anatomical characteristics and nonspecific symptoms, early diagnosis of pancreatic cancer is very rare; only 15–20% of patients undergo resection, while the remaining 80–85% of patients are diagnosed with metastatic or locally advanced disease, in which palliative therapies, such as chemotherapy and radiation, are the only treatment options 5, 6. For those patients who undergo resection, the prognosis remains poor owing to the high rate of local recurrence and/or distant metastasis. Due to the aggressive growth pattern and differing definitions of tumor involvement, microscopic involvement of the resection margin (R1) occurred in 0–83% of the resected patients; resection margin was proven to be one of the most important factors related to the prognostic outcome of patients resected for PC, positive resection margin usually resulted in higher risk of local recurrence and distant metastasis 7.

Chemotherapy and radiation therapy were applied postoperatively in the context of a multimodal approach. The adjuvant chemotherapy has been established as a standard treatment following surgical resections, based on the results of previous clinical trials (CONKO‐001 and ESPC‐1) 8, 9. However, the role of adjuvant radiotherapy in PC remains controversial owing to conflicting results from multicenter, randomized clinical trials 9, 10, 11. Compared with GITSG in 1985, EORTC and ESPC‐1 trials failed to reproduce a similar radiotherapy‐related mortality benefit. However, the recent studies have demonstrated that the adjuvant radiotherapy was associated with improved survival for resected PC, and the authors suggested that adjuvant radiotherapy might be included in the standard treatment for resected PC 12, 13.

Neoadjuvant therapy has been applied in many malignant tumors, including gastric, colorectal, breast, and pancreatic cancer. This treatment has a strong rationale and many theoretical advantages, such as inducing tumor regression, early treatment of micrometastatic lesions, reducing the risk of R1 resection and peritoneal implantation during operation, and assessment of tumor chemosensitivity in vivo 14. Numerous studies investigated the role of neoadjuvant therapy in solid malignant tumor, with some impressive and encouraging results 14, 15, 16, 17.

As mentioned previously, because of aggressive disease and low rates of early diagnosis, 30–40% PC patients present with “borderline resectable” or “locally advanced” disease when first diagnosed. These patients have a low resection rate and high possibility of R1 resection, making them theoretically the best candidates to undergo neoadjuvant therapy. Over the past 20 years, many clinical trials and retrospective studies have evaluated the safety and effectiveness of neoadjuvant chemotherapy, radiation, or chemoradiation in patients with resectable or borderline resectable, locally advanced PC. Neoadjuvant therapy is increasingly applied in patients with PC to optimize outcomes and the efficacy of neoadjuvant therapy and its indications for PC has become a hot topic in recent years. However, because of different study design, different selected patients, and variation in the chemotherapy regimens, the results of reported studies that focused on the neoadjuvant therapy in PC varied significantly; there are still controversies and no clear consensus or guideline for the use of neoadjuvant therapy in PC 7, 18.

In this study, we performed a systematic review and meta‐analysis in which we selected prospective studies for further analysis after a comprehensive review of the published literature. We sought to investigate the tumor response, toxicity, resection rate, R0 resection, histological changes, and the long‐term survival after the neoadjuvant chemotherapy, radiation, or chemoradiation in patients with PC.

## Material and Methods

### Search strategy and selection criteria

A systematic search of the published literature using Web of Science, EMBASE, Cochrane Database, MEDLINE (PubMed as the search engine), and CNKI was performed. The last search was performed in April 2015. We selected relevant literature back to January 2000. The search strategy was based on the combination of the following search key words: “pancreatic cancer/tumor/adenocarcinoma/neoplasm” and “neoadjuvant/preoperative” and “therapy/chemotherapy/radiation/chemoradiation” and “prospective/clinical trial”, with language in English or Chinese.

Relevant literatures were reviewed independently by two authors (H.X. Zhan and J.W. Xu). Titles and abstracts of articles were initially screened. Review articles, retrospective studies, case series, case reports, and conference abstracts were excluded. Repeated reports or studies that did not focus on tumor response, surgical procedure, and long‐term survival were also excluded. Full‐text articles that met the inclusion criteria were then thoroughly reviewed. H.X. Zhan and J.W. Xu used standardized data collection forms, independently extracted relevant data from the full‐text articles, and summarized the articles. Disagreements with study selection and data were resolved by discussion and consensus.

Based on the AHPBA/NCCN standard 19, the included studies were divided into three groups: (1) resectable PC before neoadjuvant therapy; (2) borderline resectable or locally advanced PC, and; (3) all types of PC. In nine studies, the authors did not describe the details of resectability criteria or assessed the resectability based on the multidisciplinary team discussion, and tumors were grouped according to the stated criteria.

### Outcomes

The primary outcome measures were tumor response and resectability. Tumor response was graded and scored according to the RECIST criteria 20: (1) Complete response (CR): disappearance of all target lesions (radiographic) or no vital tumor cells (histopathologic); (2) Partial response (PR): 30% decrease in the target lesion (radiographic) or marked signs of tumor regression (histopathologic); (3) Stable disease (SD): no change or small changes that did not meet the above criteria, and; (4) Progressive disease (PD): 20% increase in the target lesion (radiographic), or distant metastases (radiographic or histopathologic).

Histologic response to neoadjuvant therapy was graded in all resected surgical specimens using the Evans’ criteria 21: (1) Grade I: Characteristic cytological changes of malignancy are present, but little (<10%) or no tumor cell destruction is evident; (2) Grade II: In addition to characteristic cytological changes of malignancy, 10–90% of tumor cells are destroyed; IIa Destruction of 10–50% of tumor cells; IIb Destruction of 51–90% of tumor cells; (3) Grade 3: Few (<10%) viable‐appearing tumor cells are present; Grade 4: No viable tumor cells are present. Resectability rate was defined as the percentage of patients resected divided by the total number of patients who received neoadjuvant treatment. Percentage of R0 resection was calculated by dividing the total number of R0 resection patients by the total number of patients resected.

The secondary outcome measures included survival, toxicity, morbidity, mortality, and histopathologic changes. Survival time was calculated according to the methods published by Kleeff 18. Toxicity was scored using the RTOG/EORTC criteria 22. Only grade 3/4 toxicity data were collected in this study.

### Statistical analysis

Revman 5.3, MetaAnalyst Version 3.13, and SPSS 16.0 (SPSS, Inc., Chicago, IL) were used for statistical analysis. *Q*‐test was used to assess the heterogeneity among the selected studies, and the corresponding 95% confidence intervals were also calculated. The *I* ^2^ index evaluates the extent of true heterogeneity. The overall survival time was calculated by the formula mentioned in Kleeff's study.

## Results

**Figure 1:**
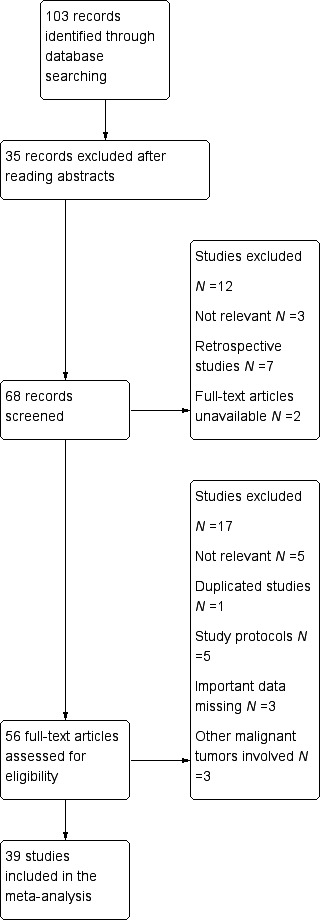
Flowchart of manuscript selection for meta‐analysis.

The systematic literature search identified 103 relevant abstracts. Forty‐seven studies were excluded after screening of the abstracts. Fifty‐six full‐text articles were reviewed, in which 17 studies were excluded due to duplicated studies, no relevant important data, or low research quality and the full‐text versions of two articles could not be obtained. In the end, 39 prospective studies occurring in the time frame of January 2000 to April 2015 were eligible to be included in this review 23, 24, 25, 26, 27, 28, 29, 30, 31, 32, 33, 34, 35, 36, 37, 38, 39, 40, 41, 42, 43, 44, 45, 46, 47, 48, 49, 50, 51, 52, 53, 54, 55, 56, 57, 58, 59, 60, 61 (Fig. 1).

**Figure 2:**
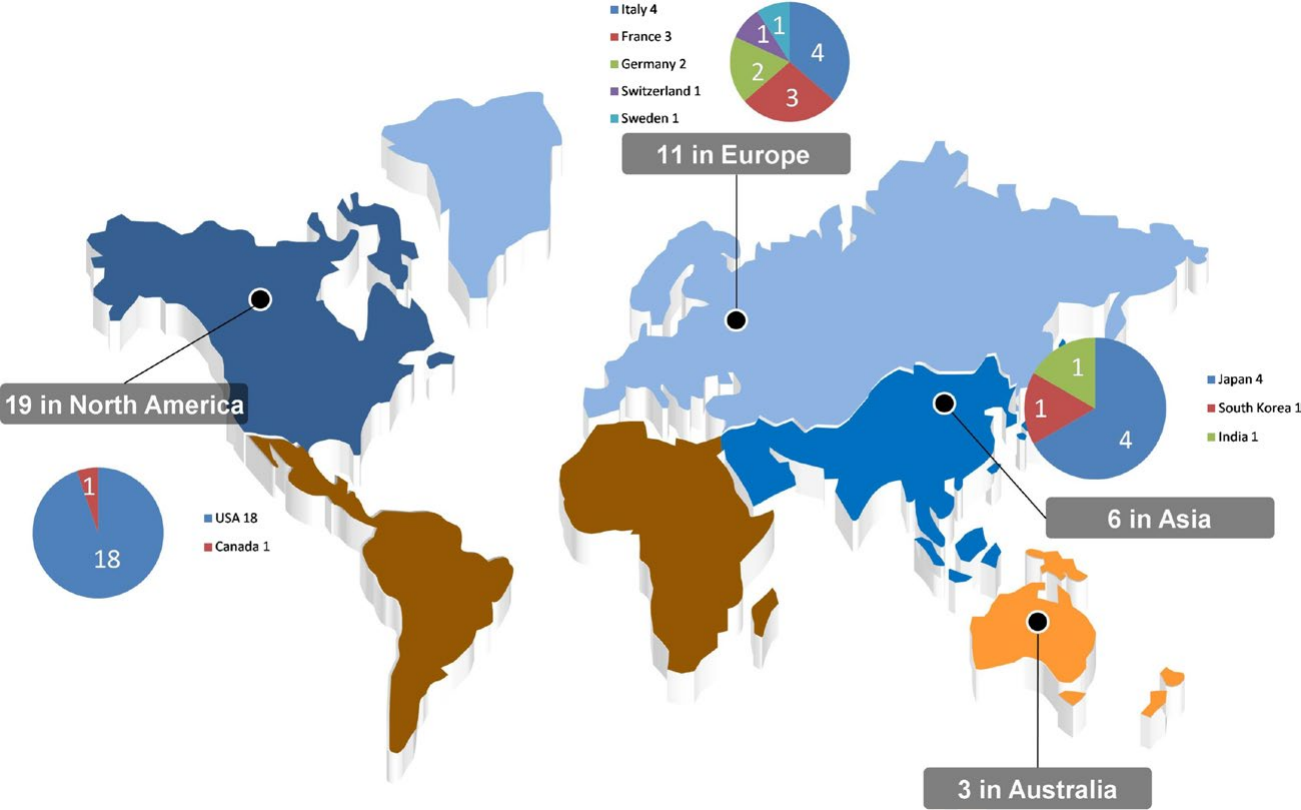
World distribution of selected studies (This map was generated from raw material in the website http://www.1ppt.com/article/2921.html. Microsoft Office Excel & Powerpoint Professional Plus 2007(Microsoft Corporation, Redmond, WA) were used to modify the picture.

These 39 studies included 1458 patients; the median number of patients per study was 37.0. Eighteen studies were published by U.S. medical centers, 11 studies from the European countries (Italy 4, France 3, Germany 2, Switzerland 1, and Sweden 1), 6 studies from Asia (Japan 4, South Korea 1, and India 1), 3 studies from Australia, and 1 study from Canada (Fig. 2, Table 1).

**Table 1:** Distribution of selected studies and patients

Group	Number of studies(%)	Number of patients involved	Patients per Study Median
Group1 (Resectable)	14 (35.9)	616	44 (10–110)
Group2 (Borderline resectable and locally advanced)	19 (48.7)	592	31 (7–53)
Group3 (Both)	6 (15.4)	250	42 (23–68)
Total	39	1458	37

There were two phase I, three phase II, and 20 phase III clinical trials, including three randomized controlled trials. Patients were divided into three groups according to the resectability criteria. Fourteen studies investigated the role of neoadjuvant therapy in patients with resectable PC (group 1, *n *= 616). Nineteen studies focused on patients with borderline and/or locally advanced PC (Group 2, *n *= 592). Six studies included all patients with PC (Group 3, *n *= 250).

### Chemotherapy agents

In one study, only preoperative radiation (no chemotherapy) was administered. In the 38 other studies, neoadjuvant chemotherapy was administered to patients with PC. In 23.07% (9/39) of the studies, patients received chemotherapy only. In 74.36% (29/39) of the studies, patients received chemoradiation therapy. Among these studies, monochemotherapy, which included gemcitabine (*n *= 6), capecitabine (*n *= 2), 5‐FU (*n *= 1), and docetaxel (*n *= 1) was used in 10 studies. Combination chemotherapy was used in 28 studies, in which a gemcitabine‐based regimen was most frequently administered (*n *= 22), followed by a 5‐FU‐based regimen (*n *= 4), including: gemcitabine + oxaliplatin (*n *= 5), gemcitabine + S‐1 (*n *= 2), gemcitabine + cisplatin (*n *= 4), gemcitabine + 5‐FU + cisplatin (*n *= 2), gemcitabine + docetaxel (*n *= 3), gemcitabine + capecitabine (*n *= 1), gemcitabine + capecitabine + docetaxel (*n *= 1), gemcitabine + bevacizumab (*n *= 1), gemcitabine + cetuximab (*n *= 1), gemcitabine + 5‐FU+ cetuximab (*n *= 1), and gemcitabine‐based combination chemotherapy (details not mentioned) in 1 study and 5‐FU + cisplatin ± cytarabine (*n *= 4). Different chemotherapy regimens were applied in two studies (gemcitabine + oxaliplatin vs. 5‐FU + irinotecan + oxaliplatin in one study and gemcitabine vs. gemcitabine + cisplatin in one study).

For the duration of chemotherapy, schedule of 6–10 weeks was the most widely used treatment plan (*n *= 15), followed by the 11–15 weeks (*n *= 10), <5 week (*n *= 8), 16–20 week (*n *= 2), 21–25 week (*n *= 2), and >25 week (*n *= 1). Borderline resectable and local advanced patients underwent longer treatment cycles than resectable PC patients (*P* < 0.05).

**Figure 3:**
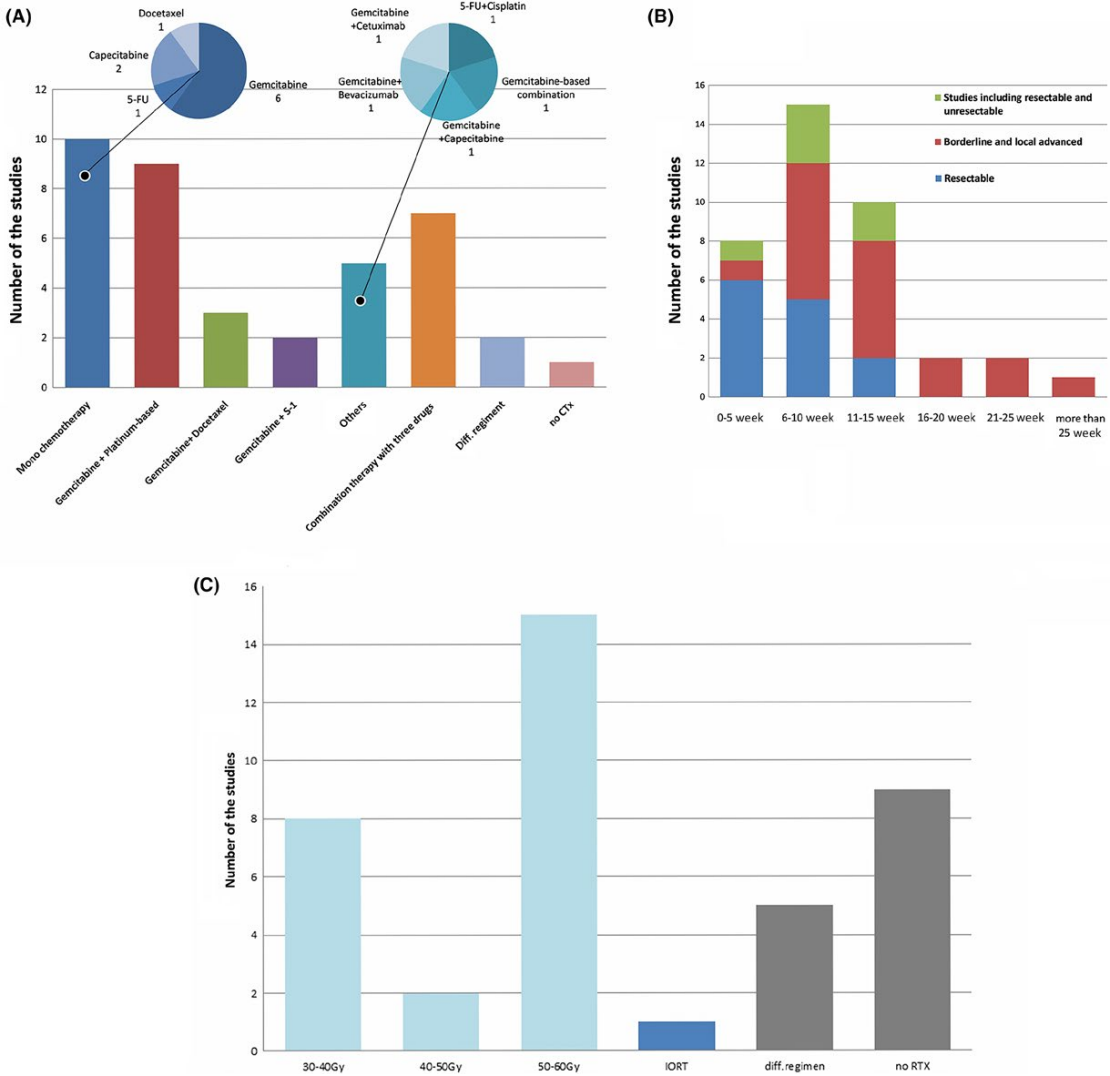
Details of neoadjuvant chemotherapy and radiation applied in PC patients. (A) Chemotherapy regimens. different drug regimen, studies comparing/using different drug regimen (*n *= 2); no CTx, no chemotherapy applied (only radiotherapy, *n *= 1). (B) Duration of chemotherapy applied in PC patients. (C) Radiation dose.

The details of the chemotherapy regimens are described in Figure 3A and B.

### Neoadjuvant radiation

Radiotherapies were administered in 76.92% (30/39) of the studies, including chemoradiation in 74.4% (29/39) studies and radiation alone in one study. Intraoperative radiation (IORT) was applied with a dose of 10 Gy in one study. Proton beam therapy with a dose of 25 Gy was used in one study. Carbon‐ion radiotherapy (30–36.8 Gy) was given in one study. The patients received doses ranging from 30 to 55.8 Gy. In these 30 studies, 45–50.4 Gy was the most frequently used (53.33%, 16/30). The applied radiation doses are summarized in Figure 3C.

### Tumor response

Evaluation of tumor response to neoadjuvant therapy was not mentioned in two studies (5.12%). The 37 other studies described the tumor response to neoadjuvant therapy based on the radiography or histological response. In 23 studies (58.97%), RECIST criteria were used to classify the tumor response. Three studies (7.69%) used the WHO classification, and the criteria were well defined. Seven studies (17.94%) evaluated the response based on the histological changes of surgically resected specimens. Four studies (10.02%) did not state well‐defined criteria for tumor response grading.

### Complete response

**Figure 4:**
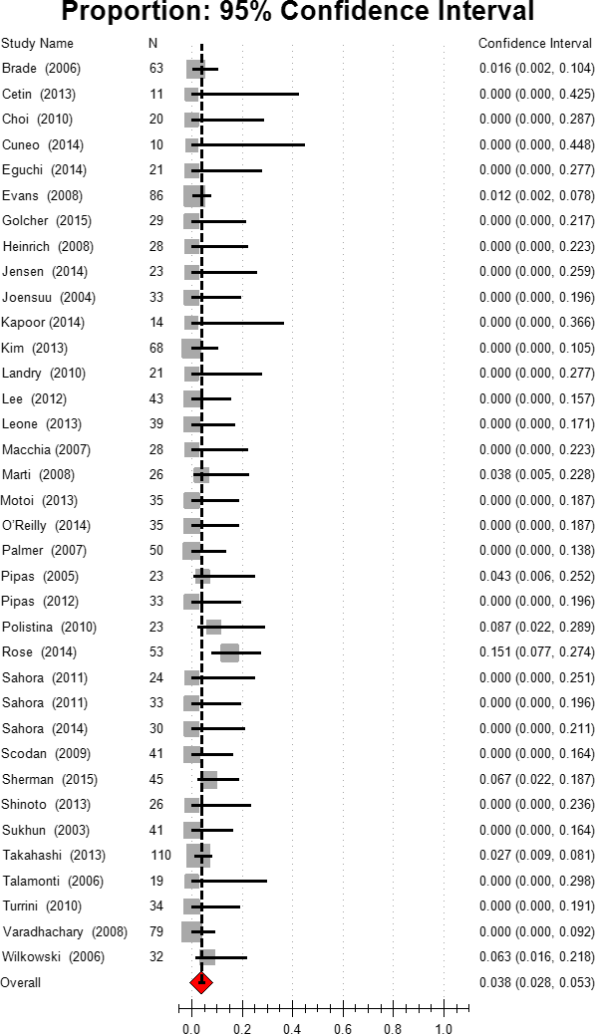
Estimated complete response.

Complete response (CR) to neoadjuvant therapy in radiography or pathology was noted in 20.51% (8/39) of the studies. The other studies reported no CR. For all patients in the selected studies, the estimated complete response was 3.80%, 95% CI [2.8, 5.3]. Based on the subgroup analysis, the CR rate was 1.8% in group 1 (95% CI [1.0–3.4]), and 4.9% (95% CI [3.1–7.7]) in group 2. The combination chemotherapy resulted in a higher CR rate (3.9%, 95% CI [2.5, 6.0]) than did gemcitabine (2.9%, 95% CI [1.5,5.7]) or other mono chemotherapy (2.0%, 95% CI [0.4–9.4]). Chemoradiation also induced a higher CR rate than chemotherapy (4.0% vs. 1.4%). Higher incidence of CR was also observed in the gemcitabine‐based chemotherapy group (Fig. 4, Table 2).

**Table 2:** Tumor response after neoadjuvant therapy

Group	Complete response	Partial response	Stable disease	Progressive disease
Total [95% CI]	3.8% [2.8,5.3] *I* ^2^ * *= 0.0% (*n *= 36)	20.9% [15.5,27.6] *I* ^2^ * *= 44.3% (*n *= 34)	54.3% [45.9,62.5] *I* ^2^ * *= 45.6% (*n *= 35)	16.0% [12.7,19.9] *I* ^2^ * *= 35.8% (*n *= 35)
Resectable [95% CI]	1.8% [1.0,3.4] *I* ^2^ * *= 0.0% (*n *= 13)	14.6% [7.5,26.4] *I* ^2^ * *= 44.3% (*n *= 12)	62.2% [46.5,75.7] *I* ^2^ * *= 45.7% (*n *= 12)	13.4% [8.4,20.7] *I* ^2^ * *= 36.0% (*n *= 12)
Borderline resectable [95% CI]	4.9% [3.1,7.7,] *I* ^2^ * *= 9.7% (*n *= 18)	28.1% [20.1,37.8] *I* ^2^ * *= 43.9% (*n *= 18)	50.5% [40.8,60.2] *I* ^2^ * *= 45.4% (*n *= 23)	17.0% [13.0,22.0] *I* ^2^ * *= 36.2% (*n *= 23)
Gemcitabine monotherapy^a^ [95% CI]	2.9% [1.5,5.7] *I* ^2^ * *= 0.0% (*n *= 6)	23.5% [6.9,56.0] *I* ^2^ * *= 48.0% (*n *= 5)	34.3% [6.3,80.2] *I* ^2^ * *= 48.8% (*n *= 5)	15.2% [7.7,27.7] *I* ^2^ * *= 39.8% (*n *= 5)
Non‐gemcitabine monotherapy [95% CI]	2.0% [0.4,9.4] *I* ^2^ * *= 0.0% (*n *= 3)	19.7% [2.9,66.4] *I* ^2^ * *= 46.9% (*n *= 3)	48.2% [24.4,72.8] *I* ^2^ * *= 42.8% (*n *= 3)	23.8% [13.9,37.5] *I* ^2^ * *= 20.8% (*n *= 3)
Combination therapy [95% CI]	3.9% [2.5,6.0] *I* ^2^ * *= 6.4% (*n *= 25)	22.7% [17.1,29.4] *I* ^2^ * *= 41.6% (*n *= 14)	57.1% [49.3,64.5] *I* ^2^ * *= 43.6% (*n *= 27)	15.4% [11.8,19.9] *I* ^2^ * *= 36.7% (*n *= 29)
Gemcitabine‐based chemotherapy^b^ [95% CI]	3.7% [2.5,5.5] *I* ^2^ * *= 9.2% (*n *= 27)	23.0% [16.6,30.9] *I* ^2^ * *= 44.5% (*n *= 25)	55.9% [46.4,64.9] *I* ^2^ * *= 45.8% (*n *= 28)	14.9% [11.2,19.4] *I* ^2^ * *= 37.7% (*n *= 28)
Non‐gemcitabine‐based chemotherapy [95% CI]	1.8% [0.6,5.0] *I* ^2^ * *= 0.0% (*n *=* *7)	20.3% [9.9,37.2] *I* ^2^ * *= 43.8% (*n *= 7)	47.9% [29.9,66.5] *I* ^2^ * *= 45.1% (*n *= 7)	20.4% [14.4,28.0] *I* ^2^ * *= 17.5% (*n *= 7)
Chemotherapy [95% CI]	1.4% [0.5,3.7] *I* ^2^ * *= 0.0% (*n *= 8)	12.7% [8.1,19.5] *I* ^2^ * *= 33.7% (*n *= 9)	60.2% [45.2,73.5] *I* ^2^ * *= 45.7% (*n *= 9)	14.5% [9.4,21.7] *I* ^2^ * *= 34.7% (*n *= 9)
Chemo + radiotherapy [95% CI]	4.0% [2.8,5.8] *I* ^2^ * *= 4.9% (*n *= 28)	23.8% [16.9,32.3] *I* ^2^ * *= 45.0% (*n *= 26)	51.8% [41.6,61.9] *I* ^2^ * *= 45.7% (*n *= 26)	16.4% [12.4,21.4] *I* ^2^ * *= 36.8% (*n *= 26)

Group labels of Table 3 and 6 are consistent with Table 2.

a We divided the patients into three groups: ① Gemcitabine monotherapy; ② other regimens monotherapy; ③ Combination therapy.

b The patients were divided into two groups: ① Gemcitabine‐based chemo (including monotherapy and combination therapy); ② other regimens chemotherapy (including monotherapy and combination therapy).

### Partial response

**Figure 5:**
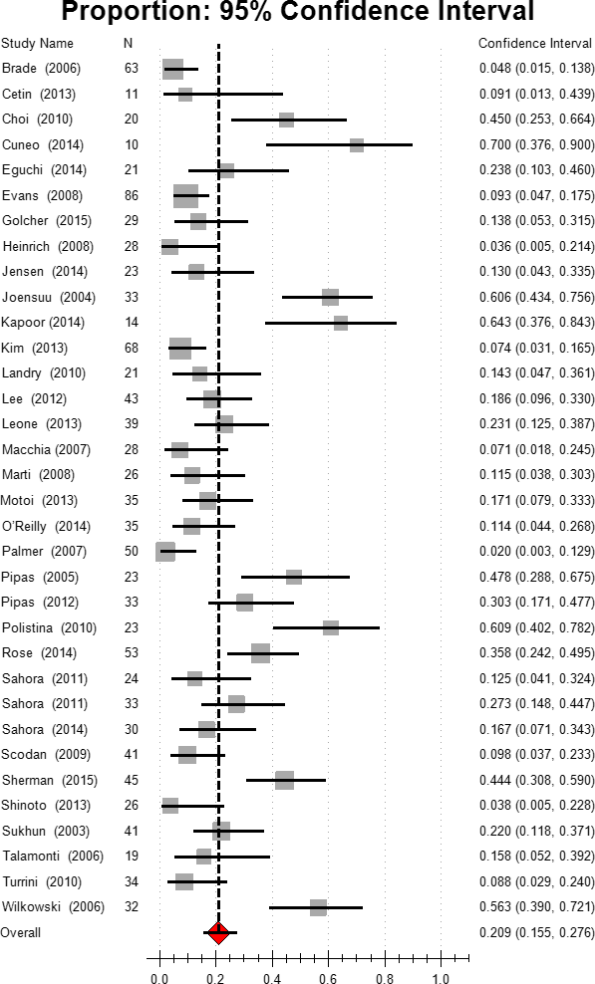
Estimated partial response.

The overall Partial response (PR) rate after neoadjuvant therapy was 20.9%, 95% CI [15.5, 27.6]. group 2 patients with advanced‐stage disease had a significantly greater PR (28.1%, 95% CI [20.1, 37.8]) than did group 1 patients with resectable PC (14.6%, 95% CI [7.5, 26.4]). The combination chemotherapy (22.7%, 95% CI [17.1, 29.4]) did not achieve better PR than the gemcitabine‐monochemotherapy (23.5%, 95% CI [6.9, 56.0]). Gemcitabine‐based chemotherapy (including both monotherapy and combination chemotherapy) resulted in a slightly better PR rate than non‐gemcitabine‐based chemotherapy group (23.0% vs. 20.3%). Chemoradiation also induced a better PR rate than did chemotherapy alone (23.8% vs. 12.7%). These details are summarized in Figure 5 and Table 2.

### Stable disease and Progressive disease

The estimated SD rate was 54.3% (95% CI [45.9,62.5]) in all patients. group 2 patients had less SD than that of group 1 patients. Chemoradiation resulted in less SD. For PD, the overall rate was 16.0%, 95% CI [12.7,19.9]. There was no significant difference between group 1 and group 2 in PD (Table 2). However, gemcitabine‐based chemotherapy resulted in less PD than did other chemotherapy regimens (14.9% vs. 20.4%). (Table 2).

### Toxicity

**Figure 6:**
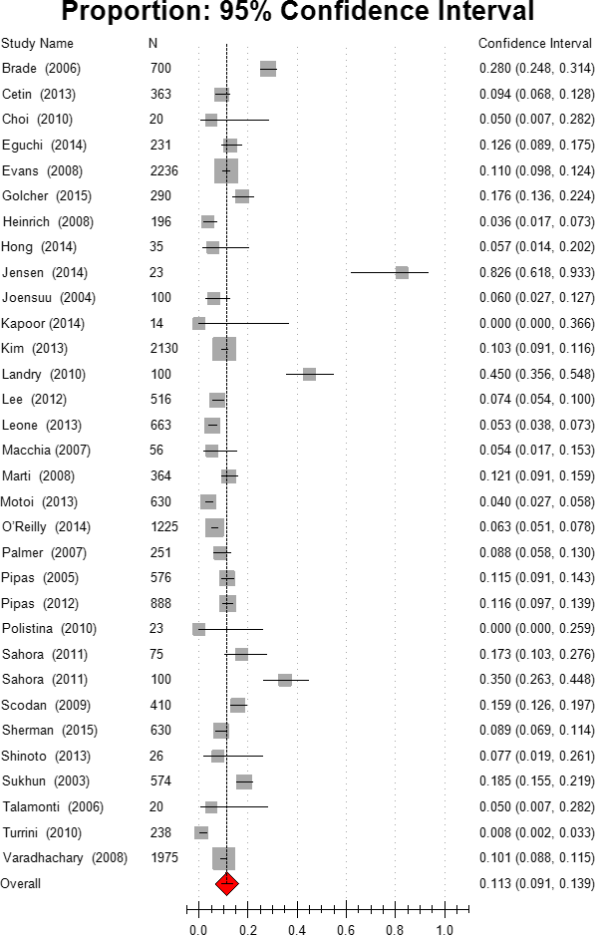
Grade 3/4 toxicity.

In the current review, only severe toxicity (Grade 3/4) data were collected and analyzed. Relevant data were available in 87.17% (34/39) of studies, in which grade 3/4 toxicity events were not reported in 5.13% of the studies (2/39). For all patients, the estimated rate of grade 3/4 toxicity was 11.3%, 95% CI [9.1,13.9]. Combination chemotherapy resulted in higher toxicity (12.3%, 95% CI [9.8,15.3]) than the toxicity observed in the monochemotherapy group (6.7%,95% CI [3.4,12.7]; Fig. 6 and Table 3).

**Table 3:** Resection rate and R0 resection after neoadjuvant therapy in pancreatic cancer

Group	Resection rate	R0 resected
Total [95% CI]	57.7% [49.5,65.5] *I* ^2^ * *= 45.8% (*n *= 39)	84.2% [80.1,87.5] *I* ^2^ * *= 27.8% (*n *= 36)
Resectable [95% CI]	73.0% [64.8,79.9] *I* ^2^ * *= 40.7% (*n *= 14)	88.2% [82.1,92.5] *I* ^2^ * *= 34.2% (*n *= 12)
Borderline resectable [95% CI]	40.2% [28.3,53.4] *I* ^2^ * *= 45.8% (*n *= 19)	79.4% [72.2,85.0] *I* ^2^ * *= 16.9% (*n *= 18)
Gemcitabine monotherapy [95% CI]	67.2% [43.3,84.6] *I* ^2^ * *= 46.7% (*n *= 6)	91.7% [80.2,96.8] *I* ^2^ * *= 39.8% (*n *= 6)
Non‐gemcitabine monotherapy [95% CI]	48.5% [26.3,71.3] *I* ^2^ * *= 45.4% (*n *= 4)	86.4% [74.5,93.2] *I* ^2^ * *= 0.0% (*n *= 3)
Combination therapy [95% CI]	55.6% [44.9,65.8] *I* ^2^ * *= 46.0% (*n *= 26)	80.5% [75.5,84.7] *I* ^2^ * *= 17.8% (*n *= 24)
Gemcitabine‐based [95% CI]	61.1% [51.1,70.2] *I* ^2^ * *= 45.9% (*n *= 28)	84.0% [78.7,88.1] *I* ^2^ * *= 33.5% (*n *= 26)
Non‐gemcitabine‐based [95% CI]	42.0% [23.2,63.4] *I* ^2^ * *= 46.2% (*n *= 8)	84.7% [76.0,90.7] *I* ^2^ * *= 0.0% (*n *= 7)
Chemotherapy [95% CI]	62.9% [45.6,77.4] *I* ^2^ * *= 45.8% (*n *= 9)	75.3% [68.6,80.9] *I* ^2^ * *= 7.3% (*n *= 9)
Chemo + radiotherapy [95% CI]	55.1% [45.4,64.4] *I* ^2^ * *= 45.9% (*n *= 29)	86.5% [82.2,90.0] *I* ^2^ * *= 23.8% (*n *= 26)

### Resection and Resection margin (R0 resection)

**Figure 7:**
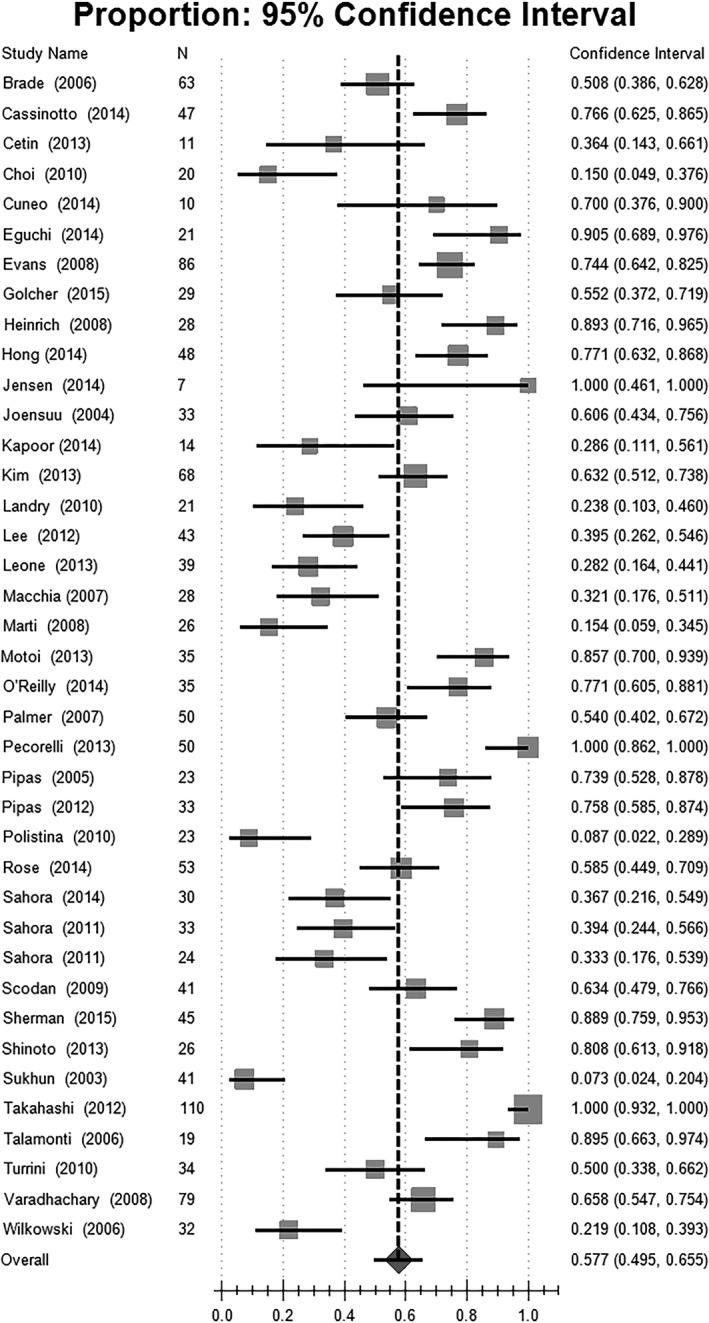
Estimated resection rate.

The resection rates after neoadjuvant therapy are shown in Figure 7 and Table 4. The surgical procedures were described in all included studies. Among the 1458 patients, 1131 underwent surgical exploration. Tumor resections were performed on 897 patients. The overall rate of resection was 57.7%, 95% CI [49.5, 65.5]. The resection rate in patients with resectable PC was 73.0%, 95% CI [64.8, 79.9]. In patients with borderline or locally advanced disease, the resection rate was 40.2%, 95% CI [28.3, 53.4]. The resection rate was higher in the gemcitabine‐based chemotherapy group than in those receiving other chemotherapy regimens (61.1% vs. 42.0%). However, combination chemotherapy did not achieve a higher resection rate than gemcitabine‐monochemotherapy (55.6% vs. 67.2%). Chemoradiation failed to achieve a higher resection rate than chemotherapy alone (55.1% vs. 62.9%).

**Table 4:** Grade 3/4 toxicity for each group

Group	Grade 3/4 Toxicity
All patients [95% CI]	11.3% [9.1, 3.9%] *I* ^2^ * *= 48.5% (*n *= 32)
Monotherapy [95% CI]	6.7% [3.4, 12.7%] *I* ^2^ * *= 48.6% (*n *= 9)
Combination therapy [95% CI]	12.3% [9.8, 15.3%] *I* ^2^ * *= 48.3% (*n *= 23)

**Figure 8:**
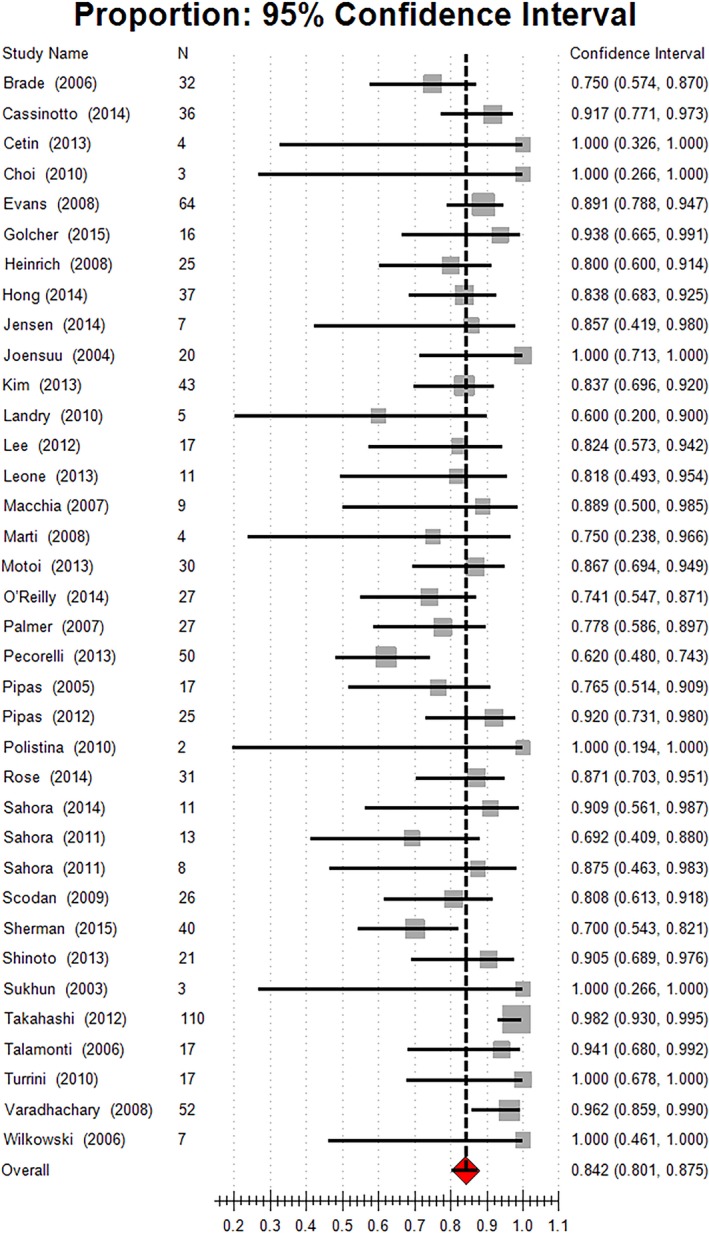
Estimated R0 resection rate.

Thirty‐six studies (92.31%) reported the resection margin status of surgical resected specimens. Margin‐negative resections (R0) were achieved in 84.2% of the resected patients, 95% CI[80.1,87.5]. The estimated proportion of R0 resections was higher in resectable patients than in patients with borderline and locally advanced disease (88.2% vs. 79.4%). Combination chemotherapy (80.5%, 95% CI [75.5, 84.7]) did not achieve better R0 resection than monochemotherapy. Gemcitabine‐monochemotherapy (91.7%, 95% CI [80.2, 96.8]) achieved the highest resection status of all chemotherapy regimens. Chemoradiation also induced a better R0 resection rate than chemotherapy alone (86.5% vs. 75.3%) (Fig. 8, Table 4).

### Histological response

Twenty‐three (58.97%; 23/39) studies described the histological changes of surgical specimens after neoadjuvant therapy. Pathological complete response (pCR) was observed in seven studies. Ten patients achieved pCR (estimated 3.2%, 95% CI [1.9,5.4]). According to the Evans’ criteria, treatment effect I/II/III/IV varied from 0% to 69.56% among these studies. Grade II was the most common treatment effect, occurring in 34.1% of patients (95% CI [24.7, 45.0]), followed by 17.8% of patients achieving grade 1 and 10.6% of patients achieving Grade 3 treatment effects. The details are summarized in Table 5.

**Table 5:** Treatment effects graded by Evans’ criteria

Treatment effect	Number of studies	Estimated percentage of treatment effect (%)	*I* ^2^	95% CI
I	15	17.8	0.425	11.8–25.9
II	14	34.1	0.447	24.7–45.0
III	12	10.6	0.340	6.9–15.9
IV	15	3.2	0.000	1.9–5.4

### Morbidity and mortality

Data regarding operation‐related morbidity and mortality were available in 51.28% (20/39) of the studies. The incidence of morbidity ranged from 0% to 75%. The estimated morbidity rate was 21.0%, 95% CI [15.3, 28.3]. Pancreatic fistula occurred in 29 patients, with its estimated incidence of 6.9%, 95% CI [4.6, 10.4]. Thirteen patients died after surgery among 1131 patients who underwent operations.

### Survival analysis

Estimated median survival times were calculated as described in the methods. Survival data were available in 89.74% of studies (35/39). Survival times were calculated from the time of diagnosis/start of neoadjuvant therapy in 30 trials and from surgery/resection in 0 trials. No detailed information regarding survival or survival calculations were provided in nine studies. The estimated median survival for all patients was 16.79 months (95% CI [9.4, 32.5], *n *= 1164). Patients who underwent surgical resections after neoadjuvant therapy had a much better prognosis than did those whose tumors were not resected (24.24 vs. 9.81 months). For patients with resectable disease before neoadjuvant therapy, the overall survival time was 17.76 months (95% CI [9.4, 27.2], *n *= 551). Patients who were diagnosed with resectable disease before neoadjuvant therapy achieved the highest survival time after surgical resections (25.29 months, 95% CI [11.7, 34.0], *n *= 361). For patients with advanced disease, the median survival time was shorter (total: 16.20 months, resected: 21.80 months, unresected: 12.16 months). Gemcitabine‐based chemotherapy resulted in a better prognosis than did other chemotherapy regimens (18.36 vs. 12.93). Chemoradiation failed to achieve a better prognosis than chemotherapy alone (16.73 vs. 16.8). (Table 6).

**Table 6:** Estimated median survival for each group

	Estimated media*n* survival (*m*_p_)		
Group	Total (range)	Resected (range)	Unresected (range)
Total	16.7 (9.4–32.5) (*n *= 33)	24.24 (11.7–47.4) (*n *= 19)	9.81 (7.1–17) (*n *= 16)
Resectable	17.76 (9.4–27.2) (*n *= 14)	25.29 (11.7–34) (*n *= 7)	8.82 (7.1–11) (*n *= 6)
Unresectable	16.2 (10.6–32.5) (*n *= 17)	21.8 (15–32) (*n *= 9)	12.16 (8.7–17) (*n *= 7)
Gemcitabi*n*e monotherapy	19.18 (10.6–25) (*n *= 4)	34 (34) (*n *= 1)	7.1 (7.1) (*n *= 1)
*Other regimens* monotherapy	14.77 (11.3–17.3) (*n *= 3)	24.12 (15–32) (*n *= 4)	9.73 (8.7–11) (*n *= 3)
Combination therapy	16.87 (9.4–32.5) (*n *= 24)	22.71 (11.7–47.4) (*n *= 13)	10.9 (8.5–17) (*n *= 11)
Gemcitabine‐based chemo	18.36 (10.6–32.5) (*n *= 24)	26.21 (16.3–47.4) (*n *= 12)	10.15 (7.1–17) (*n *= 10)
*N*o*n‐*gemcitabine‐based chemo	12.93 (9.4–17.3) (*n *= 7)	19.21 (11.7–32) (*n *= 6)	9.23 (8.5–11) (*n *= 5)
Chemotherapy	16.8 (13–27.2) (*n *= 8)	24.33 (16.3–34.7) (*n *= 5)	10.7 (8.6–13.2) (*n *= 5)
Chemo + radiotherapy	16.73 (9.4–32.5) (*n *= 24)	24.21 (11.7–47.4) (*n *= 14)	9.47 (7.1–13) (*n *= 11)

## Discussion

Multidisciplinary treatment (MDT) has been recommended for PC^62^. New treatment strategies have been developed to improve the prognosis of patients with PC. Among these strategies, neoadjuvant therapy has received substantial recent attention due to its potential advantages 6, 14, 62, such as down‐staging tumors and increasing total and R0 resection rates 14. However, the clinical value of neoadjuvant therapy remains controversial. Thus, a systematic review and meta‐analysis focusing on the prospective studies examining neoadjuvant therapy in PC was performed in order to comprehensively evaluate the clinical outcomes of neoadjuvant therapy. We showed that patients with borderline or locally advanced tumors might benefit from neoadjuvant therapy, while neoadjuvant therapy has not been proven to be beneficial and should be considered with caution in patients with resectable PC.

Many recent retrospective and prospective studies have focused on neoadjuvant therapy in PC 19, 24, 30. However, the clinical value of this approach is still unclear, due to the limitations of small sample sizes, lack of control groups, ambiguous definitions of resectability, and various regimens of therapy. Gillen and colleagues performed a systematic review and meta‐analysis in 2010, in which they comprehensively illustrated the pros and cons of neoadjuvant therapy 18. However, Gillen's review included prospective trials as well as retrospective studies, which weakened the strength of evidence. Only prospective trials were included in our review, reducing the heterogeneity and furtherly improving the quality of the study and the levels of evidence. In addition, the quality of clinical trials has dramatically improved in the past 5 years; some prospective, randomized trials were performed and reported during that period 36, 54. Moreover, since the consensus regarding the definitions of borderline resectable/unresectable tumors has been reached 7, 19, the ability to compare the results of various trials has furtherly improved. Therefore, it is the time now to reevaluate the value of neoadjuvant therapy in PC.

The indications of neoadjuvant therapy remain controversial. Which patients can benefit from neoadjuvant therapy is still unknown. In the group of patients with resectable tumors before neoadjuvant therapy, 73.0% underwent resection, and the R0 resection rate was 84.2% after neoadjuvant treatment. This finding was similar to the previous literature 63, 64, which reported a 78–96% resection rate in patients with resectable tumors that were directly explored without neoadjuvant treatment. The median survival time was 17.76 months, which was similar to that reported in earlier studies 9, 65. Additionally, in a prospective randomized phase II trial performed by Golcher and colleagues, 245 patients with gemcitabine/cisplatin in resectable pancreatic cancer had a 4.33‐month improvement in median overall survival (mOS) after neoadjuvant chemoradiation. This result indicated that neoadjuvant chemoradiation was safe with respect to toxicity, perioperative morbidity, and mortality compared with immediate surgery. After tumor resection, the R0 resection rate changed from 48% to 52% (*P *= 0.81), and mOS improved from 18.9 to. 25.0 months (primary surgery vs. neoadjuvant chemoradiotherapy followed by surgery, *P *= 0.79). Overall, neoadjuvant treatment for patients with resectable tumors did not result in the expected values. Thus, the trial was terminated early due to slow recruiting of patients and insignificant results 60. Overall, neoadjuvant treatment for patients with resectable tumors seemingly did not show the expected clinical superiority. Considering the potential risk for tumor progression during neoadjuvant therapy, and additional invasive diagnostic methods required for histological diagnosis, neoadjuvant treatment should be considered with caution for patients with resectable tumors. However, tumor biology in some patients with resectable PC was very aggressive, these patients may present with progressive disease during the neoadjuvant therapy process, they may not benefit from surgery; neoadjuvant therapy can be beneficial for these patients and help avoid unnecessary surgery. Controversies still exist in the application of neoadjuvant therapy in resectable PC. A recent published study by Mokdad et al. 66 reported that neoadjuvant therapy followed by resection has a significant survival benefit compared with upfront resection in early‐stage, resected pancreatic head adenocarcinoma (median survival 26 vs. 21 months), but some experts argued that this survival benefit can be explained by immortal time bias 67. More powerful, prospective clinical trials should be performed to identify the value of neoadjuvant therapy in patients with resectable PC.

In patients with borderline or unresectable tumors, 40.2% underwent resection; with the R0 resection rate at 79.4% after neoadjuvant treatment. Compared with patients who underwent initial resection for locally advanced tumors 68, higher total and R0 resection rates were observed after neoadjuvant treatment. Furthermore, neoadjuvant treatment increased the overall survival time. The patients who underwent resection after neoadjuvant therapy presented with a median survival of 21.8 months, which was within the range of patients with PC with primary resection 18. In a single‐arm phase II study performed by Motoi et al., neoadjuvant chemotherapy with gemcitabine and S‐1 was administered every 21 days for two cycles to patients with resectable and borderline pancreatic cancer. The results showed that neoadjuvant chemotherapy with gemcitabine and S‐1 was well tolerated and safe when used for patients with resectable and borderline pancreatic cancer. Thirty‐five patients were eligible for the trial, R0 resection was performed for 87% in resection, and the morbidity rate (40%) was acceptable. Patients who underwent operations after neoadjuvant chemotherapy (*n *= 27) showed an increased median overall survival (34.7 months) compared with those who did not undergo resection (*P *= 0.0017) 46. In conclusion, nearly one‐third of the patients initially staged as borderline or locally advanced tumors could be completely resected after neoadjuvant treatment with an improved overall survival rate. This result proves that neoadjuvant therapy might benefit patients with borderline or locally advanced tumors, but sample size of this study is very small. Because of its high toxicity, tolerability concerns, and unpopularity, S‐1 was not applied in neoadjuvant therapy or adjuvant therapy in the treatment of PC in western countries; there was no published literature focused on S‐1 in PC from other countries besides Japan. The effect of S‐1 in neoadjuvant therapy of PC should be evaluated sufficiently worldwide. Controversies still exist, in which if technical options of resections are possible, the ISGPS does not recommend neoadjuvant therapy regimens for patients with isolated venous involvement in borderline resectable tumors. Even if arterial involvement is seen at imaging, initial surgical exploration is strongly recommended 7.

Neoadjuvant therapy seemed to be useful for selected patients, but the total CR and PR were similar to that of the patients with metastatic pancreatic cancer who underwent palliative treatment. The total CR and PR of neoadjuvant therapy were 3.8% and 20.9%, respectively. However, Conroy et al. indicated that for patients with metastatic pancreatic cancer, the CR and PR of FOLFIRINOX were 0.6% and 31%, and gemcitabine were 0% and 9.4% 69. Nevertheless, some regimens demonstrated better therapeutic effects. The gemcitabine‐based regimens showed superior CR and PR compared with other regimens. The CR and PR of chemoradiotherapy were higher than those of chemotherapy. Interestingly, a higher CR or PR did not indicate a higher resection rate or longer survival.

Several factors may partly explain this paradox. The current criteria for evaluation of response rate are based on imaging examination and laboratory tests 20, 42, which may not completely reflect the treatment effects of neoadjuvant therapy. Especially for patients with resectable tumors, in which significant tumor shrinkage is not always observed 70. Lower CR and PR for patients with resectable tumors were also observed in our study. Some newly developed criteria, such as histological evaluation, are considered more effective and accurate for assessing the effects of chemotherapy and neoadjuvant therapy 31. However, the correlations between the grade of histological response and resection rate and overall survival should be explored further.

Various treatment regimens were mentioned in the current review, including gemcitabine monotherapy, 5‐FU monotherapy, gemcitabine‐based combination therapy, and chemoradiotherapy. Although a comprehensive systematic review was performed, the best regimen for neoadjuvant therapy is still unknown. Some regimens showed advantageous resection and R0 rates, but the limitations of these treatments attenuated the clinical values. For instance, we found that gemcitabine monotherapy had a higher resection rate and R0 rate than combination therapy. However, many clinical trials have confirmed the superior effects of combination chemotherapy 69, 71, 72. The existing selection bias of neoadjuvant therapy regimens may partly account for this unexpected result. Monochemotherapy is more likely to be administered to patients with resectable tumors, while combination therapy is always applied in advanced diseases. We analyzed tumor response in all patients, including resectable, borderline resectable, or metastatic diseases, while the FOLFIRINOX trial was only applied in metastatic pancreatic cancer 69. As mentioned previously, the current criteria for evaluation of response rate is based on imaging examination; however, obvious tumor shrinkage is not always observed in resectable PC. This cause may also partly explain the higher PR rate in combination therapy in metastatic patients. A recent study confirmed that FOLFIRINOX is a valuable treatment option in the neoadjuvant therapy of local advanced PC, seemed to be the most effective protocol resulting in a significantly better resection rate and overall survival than other treatments 73. Resection rates following FOLFIRINOX were 61% compared with 46% after gemcitabine and radiation. Three‐year survival rate was also better in FOLFIRINOX group than gemcitabine group (28.1% vs. 23.2%) 73. Meta‐analysis revealed that median overall survival of patients with locally advanced pancreatic cancer treated with FOLFIRINOX was better than that reported with gemcitabine (24.2 vs. 6–13 months), the resection rate and R0 resection rate were 25.9% and 78.4% in patients with local advanced PC after FOLFIRINOX therapy 74. Patients with borderline resectable or unresectable PC achieved satisfactory resection rate and R0 resection rate after neoadjuvant FOLFIRINOX‐based therapy 75. Besides FOLFIRINOX, nab‐paclitaxel plus gemcitabine was also applied as neoadjuvant therapy in patients with borderline resectable PC; the initial results showed that neoadjuvant nab‐paclitaxel plus gemcitabine therapy was safe and feasible 76, the clinical benefit should be further investigated, more clinical trials are urgently needed to confirm the best protocol for neoadjuvant therapy in patients with PC.

As a systematic review and meta‐analysis, this study had limitations. First, the ambiguous definition of resectability partly attenuates the reliability of results. Although most included trials were published in the last 10 years, which suggests reliable results, we should dialectically assess these findings. Encouragingly, some consensus regarding the definition of resectability has been reached 7, 77. This consensus may improve the quality and comparability of future treatment trials. Second, because of high variation between the involved studies, such as different regimens and duration applied, different baseline of selected patients, and long study period span, selection bias and other factors may have important influence on the statistical results. We should read these conclusions discerningly. Meanwhile, most of the included studies in our study just had one single‐arm. The lack of a control group limited our ability to effectively evaluate the clinical outcomes of neoadjuvant therapy. We cannot perform further statistical analysis between different groups and give the *p* value due to the raw data. For instance, we cannot conclude that CR rate in combination chemotherapy group is significantly higher than monochemotherapy because of the overlap of 95% CI and lack of *P* value. To some extent, we just observe the treatment benefit tendency in our study. Finally, the most effective neoadjuvant therapy regimen was not identified through our review, we expect more rigorous, and two‐arm studies to further evaluate the values of neoadjuvant therapy in PC.

In conclusion, our review indicated that neoadjuvant therapy has not been proven to be beneficial and should be considered with caution in resectable PC patients. However, neoadjuvant therapy may be beneficial to patients with borderline or local advanced tumors. Many controversies still exist, thus more rigorous, randomized, and controlled studies should be performed to comprehensively assess the indications and effects of neoadjuvant therapy.

## Conflict of Interest

None declared.
